# Evaluation of single-port robotic initial treatment of hernias (ESPRITH study): an initial case series following the IDEAL framework

**DOI:** 10.1007/s00464-025-12436-3

**Published:** 2025-11-23

**Authors:** Francesco Brucchi, Annabelle De Troyer, Filip Muysoms

**Affiliations:** 1 University of Milan, Via Festa del Perdono, 7, 20122 Milan, Italy; 2 Department of General Surgery, Ziekenhuis Aan de Stroom (ZAS) Antwerp, Antwerp, Belgium; 3 Department of General Surgery, AZ Maria Middelares, Ghent, Belgium

**Keywords:** Single-port robotics, Da Vinci SP, Inguinal hernia, Ventral hernia, IDEAL framework, Case series

## Abstract

**Background:**

The Da Vinci Single Port has received the CE marking for General Surgery in Europe in 2024. However, its role in hernia repair is largely unexplored. In this study, we present an IDEAL Stage 1 case series evaluating the Da Vinci SP system for hernia repair.

**Methods:**

We report the first European case series of SP robotic hernia repair (Da Vinci SP system, Intuitive Surgical). Consecutive patients undergoing inguinal hernia repair (IHR) with concomitant umbilical hernia, or midline ventral hernia repair (EHS M1–M4), were included between October 2024 and July 2025. Inguinal repairs (TAPP/TEP) were performed via the umbilical defect, while ventral repairs employed a suprapubic extraperitoneal access. The primary outcome was intraoperative and postoperative complications; secondary outcomes included operative time, hospital stay, and 1-month morbidity.

**Results:**

Twenty-two patients were treated (14 ventral, 8 inguinal with concomitant umbilical hernia). Median operative time was 87 min for IHR and 150 min for ventral repairs. No intraoperative complications, conversions, or additional ports were required. Median length of stay was 19.5 h; six patients (27%) required an unplanned overnight stay for pain management. Two minor complications (seroma, scrotal edema) occurred at 1-month follow-up; no recurrences or readmissions were observed.

**Conclusions:**

This IDEAL Stage 1 case series demonstrates the technical feasibility of single-port (SP) robotic hernia repair in selected patients with inguinal and ventral hernias, with no intraoperative or early postoperative complications observed. These preliminary findings support further prospective IDEAL Stage 2–3 studies with larger cohorts, standardized techniques, and long-term follow-up to validate safety and comparative outcomes.

Hernia repair is one of the most frequently performed procedures in general surgery, with a steadily growing proportion managed through minimally invasive surgery (MIS). Compared to open repair, MIS is associated with reduced wound morbidity, faster recovery, and shorter length of stay. Early MIS approaches to ventral hernias, such as laparoscopic intraperitoneal onlay mesh (IPOM), were widely adopted but raised concerns related to mesh–viscera interactions, including adhesions, chronic pain, and enteric injury [1–3]. This has driven a paradigm shift toward extraperitoneal mesh placement in the preperitoneal or retromuscular planes, which is now recommended by international guidelines for its favorable risk profile and potential long-term benefits.

Techniques such as TAPP, TEP, eTEP, PeTEP, and TAR allow for extraperitoneal placement, but remain technically demanding when performed laparoscopically due to limitations in instrument articulation and visualization, particularly for anterior and cranial dissections. The introduction of robotic multi-port systems has expanded the feasibility of these reconstructions, enabling precise dissection, intracorporeal suturing, and complex repairs such as TAR in a minimally invasive setting. Robotic abdominal wall surgery has therefore become increasingly adopted, especially for ventral and incisional hernias.

Most robotic techniques rely on multi-port access. The Da Vinci Single Port (SP) platform (Intuitive Surgical, Sunnyvale, CA) was designed to perform surgeries with one single incision by introducing a flexible 3D camera and three wristed instruments through a single 27-mm cannula [4]. Initially validated in urology and head-and-neck surgery, the SP system has recently been approved for general surgery in Europe. Early reports across colorectal and hepatobiliary surgery highlight feasibility and safety, yet its application to abdominal wall hernia repair remains largely unexplored [5]. Potential advantages include the ability to exploit existing defects (e.g., umbilical hernia) or cosmetically favorable access routes (e.g., suprapubic incision), thereby reducing the number of incisions while maintaining access to anatomical planes critical for extraperitoneal mesh placement.

Within the IDEAL framework for surgical innovation, early experiences (Stages 1–2a) are crucial for systematically documenting feasibility, safety, and short-term outcomes before progressing to comparative studies [6]. Our group previously reported pre-IDEAL investigations using cadaveric models and porcine training to establish the technical feasibility of SP approaches for abdominal wall surgery, specifically validating extraperitoneal access routes, spatial constraints, and instrument maneuverability in controlled experimental conditions. The present clinical series represents the translational extension of that preclinical work [7]. Building on this foundation, we present here an IDEAL Stage 1 clinical case series, reported in line with the PROCESS guidelines [8], evaluating the Da Vinci SP system for hernia repair. Specifically, we report outcomes in two patient groups: (i) inguinal hernias with concomitant umbilical hernia, and (ii) midline ventral hernias classified as M1–M4 by the European Hernia Society (EHS) [9]. This study aims to provide the first clinical evidence on the safety, feasibility, and early postoperative outcomes of single-port robotic hernia repair in a European center.

## Methods

### Study design and setting

This study was conducted at the Department of Surgery, Maria Middelares Hospital (Ghent, Belgium). Using a single-center observational cohort design, data from a prospectively maintained database were retrospectively analyzed. The study included all consecutive patients who underwent robot-assisted hernia repair using the Da Vinci SP system between October 2024 and July 2025. All patients provided written informed consent prior to inclusion in the study and for the surgical procedures performed. The procedures were performed exclusively by one experienced robotic abdominal wall surgeon using the SP platform for selected cases of inguinal hernia with concomitant umbilical hernia, and for ventral M1–M4 hernia repairs. Before starting the clinical application of the Da Vinci SP platform, training with the SP platform was completed using simulation, dry/wet laboratories, and case observations [7]. This case series has been reported in line with the PROCESS Guideline [8].

### Participants

Eligible participants were adults (≥ 18 years old) who either presented with the following:

- An inguinal hernia, either unilateral or bilateral, in association with a primary or incisional umbilical hernia of maximum 2 cm in diameter, or
- A midline ventral hernia classified as M1–M4 according to EHS classification with or without concomitant diastasis of the rectus abdominis muscles [9].

Exclusion criteria:

- For inguinal hernia patients: recurrence after previous preperitoneal mesh repair, prior abdominal prostatectomy, inguinoscrotal hernias.
- For ventral hernias: recurrences after previous ventral hernia repair with mesh

Patients were included consecutively.

### Surgical technique

**Fig. 1:**
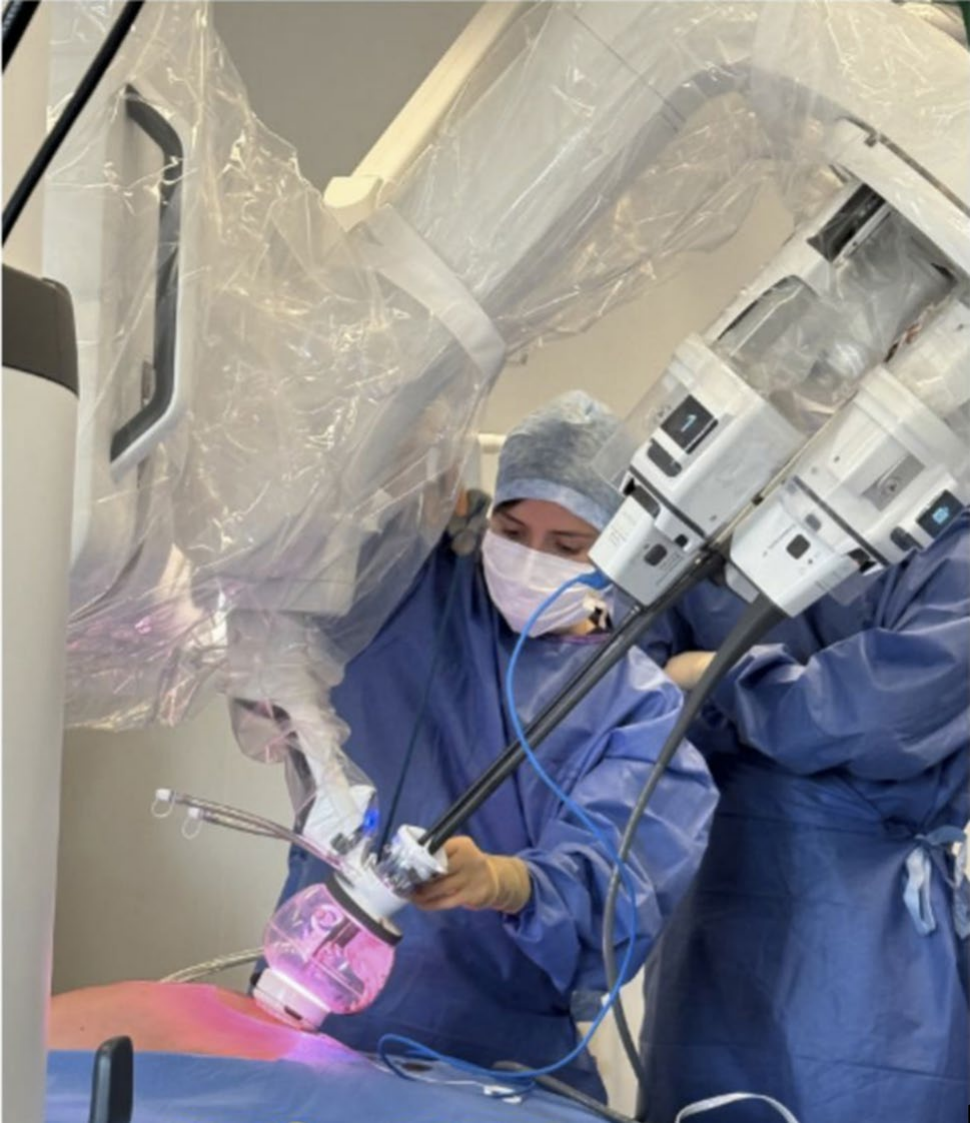
Da Vinci SP robotic platform docked and in use during a single-port suprapubic eTEP procedure

The Da Vinci SP technology is well described in other recently published papers (Fig. 1) [4, 5, 7, 10, 11]. The surgical technique used (trans-abdominal versus extraperitoneal), the mesh used (self-fixating mesh versus non-self-fixating with suture fixation), and the mesh position (retrorectus versus preperitoneal) used was deliberately variable across the cases to explore the feasibility and performance of different options. All procedures were performed under general anesthesia. In cases of minimally invasive inguinal hernia repair (IHR), no prophylactic antibiotics were administered, whereas patients undergoing SP^2^ eTEP received a single preoperative dose of antibiotic prophylaxis, as this approach was considered a semi-open procedure. Patients were instructed to void the bladder preoperatively.

All operations were carried out using the Da Vinci SP system. Surgical approaches varied based on hernia type and included TAPP, TEP for inguinal hernias, and eTEP, PeTEP, and eTEP-TAR, for ventral hernias as clinically indicated.

### Inguinal hernia repair (IHR)

Access to the abdominal cavity (for TAPP) or the preperitoneal space (for TEP) in inguinal hernia cases with concomitant primary or incisional umbilical hernia was achieved through the umbilical defect, using it as the entry point for the Da Vinci SP small access port.

The procedure begins with a 27-mm umbilical skin incision, followed by blunt and diathermic dissection of the subcutaneous tissue along the umbilical stalk. The umbilical hernia is carefully isolated and reduced into the abdominal cavity. Adequate exposure of the anterior aponeurosis is ensured to facilitate straightforward closure at the end of the operation. A blunt digital preperitoneal dissection is performed peri-umbilically. If the peritoneum could be kept intact during this dissection, a TEP approach is performed, while if that is not possible a TAPP approach is done.

The fascia is elevated, allowing for the tangential insertion of the SP small access port through the abdominal wall. The patient is then placed in a 12° Trendelenburg position to enhance visualization of the surgical field. After establishing pneumoperitoneum in TAPP cases, an initial exploratory laparoscopy is performed, including evaluation of the contralateral groin in unilateral cases to assess the need for bilateral repair.

**Fig. 2:**
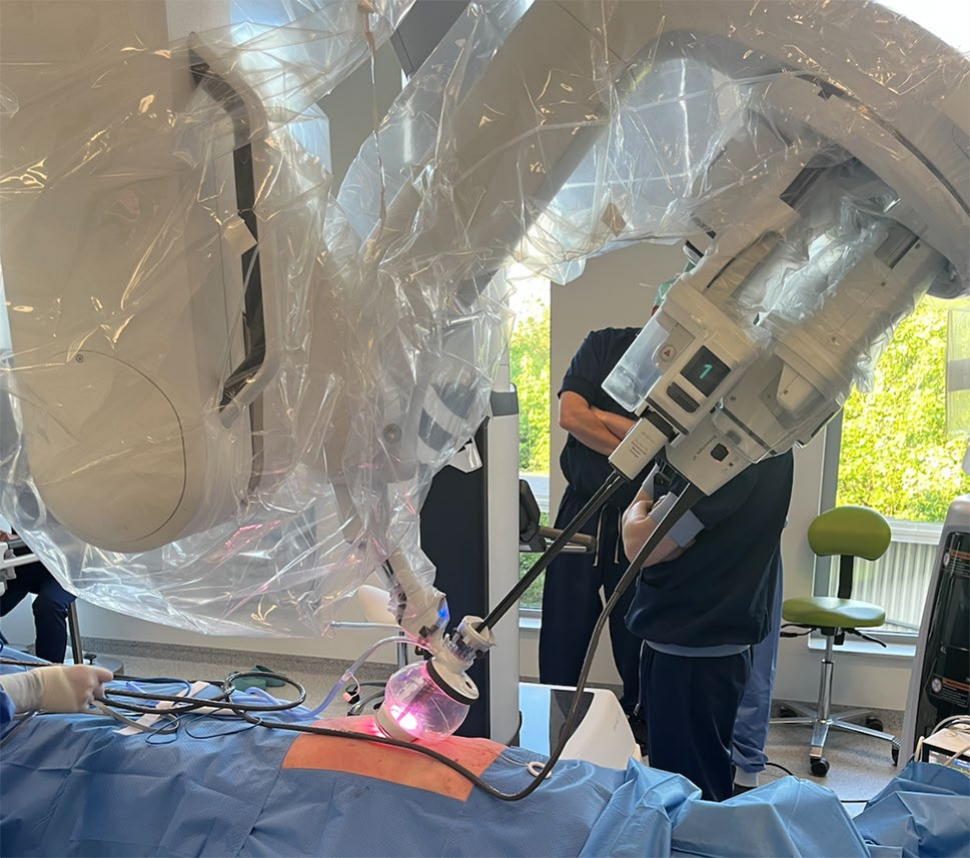
Da Vinci SP robotic platform docked and in use during a single-port TAPP procedure

The robotic cart is docked over the patient’s flank, without a mandatory docking side, thanks to the SP system’s capacity for 355° rotation around the single port. The robotic single arm is attached to the access port, and a 3D articulating endoscope is introduced through the inferior lumen. Working instruments—monopolar curved scissors on the right and fenestrated bipolar forceps on the left—are deployed through their respective lumens (Fig. 2).

Pneumoperitoneum is established, and the camera and instruments are fully operational. The SP platform’s double-jointed instruments and ergonomic design allow precise dissection in confined spaces. The system includes two additional working channels in the port access balloon to optimize assistant support throughout the procedure.

The inguinal hernia repair, whether performed using the TAPP or TEP technique, is completed in accordance with the Ten Golden Rules published by Claus et al. and by adhering to the principles of an adequate Critical View of the Myopectineal Orifice (MPO) [12, 13].

**Fig. 3:**
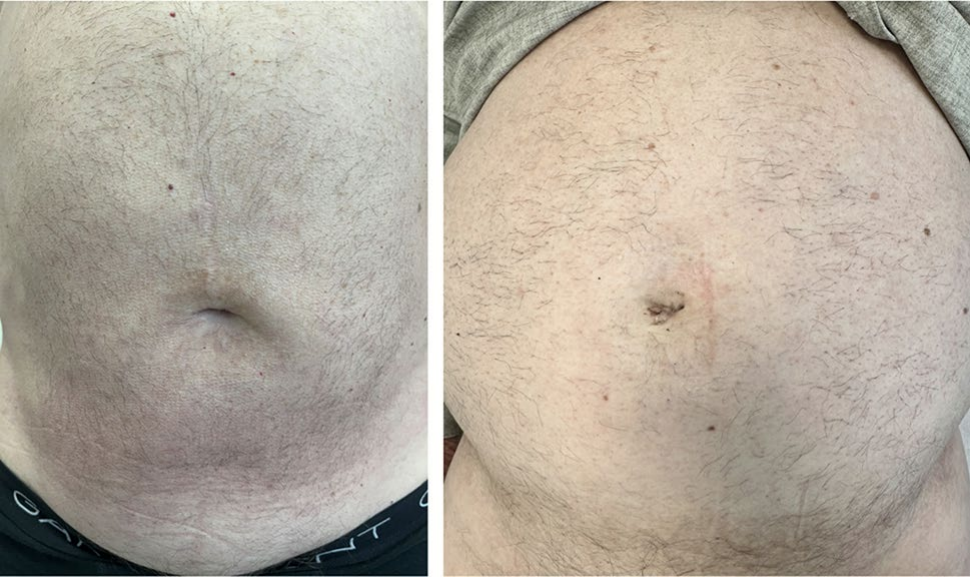
Cosmetic outcome at 1-month follow-up after inguinal hernia repair performed with the Da Vinci SP system

Upon completion of the repair, the robotic arm and cannula are withdrawn. The umbilical fascia defect is closed using a Monomax 2/0 slowly absorbable continuous 4-hydroxybutyrate suture for smaller primary hernias. For secondary incisional hernias or hernias larger than 2 cm, a mesh repair was done either preperitoneal or retromuscular. Thereafter, a layered closure of the subcutaneous tissue with polyglactin sutures (Vicryl 3/0) and skin with polyglecaprone (4–0 Monocryl) subcuticular sutures was done ensuring a cosmetically favorable result (Fig. 3).

### Ventral hernia repair: single-port suprapubic eTEP (SP.^2^ eTEP)

The patient is positioned supine with hips hyperextended at the level of the anterior superior iliac spina (ASIS) with the table flexed 12–15 degrees and a head-down tilt. Arms are tucked alongside the patient.

The procedure utilizes the Da Vinci SP small access port, along with a monopolar curved scissors, fenestrated bipolar forceps, and a needle driver. A horizontal 2.7-cm midline suprapubic incision, approximately 2 cm above the pubic symphysis, is made via a “mini-Pfannenstiel” technique. The subcutaneous tissue is bluntly dissected to expose the anterior rectus fascia. The anterior rectus fascia is opened horizontally exposing the rectus abdominis muscles. The rectus muscles are split bluntly on the midline vertically to gain access to the preperitoneal plane in Retzius space. This space is further developed bluntly using finger dissection, aided by a Raytec gauze, to create sufficient working space for the placement of the small access port. This dissection is easily performed in the retropubic space and cranial up to the level of the arcuate lines, provided there was no previous infraumbilical surgery like cesarean section.

**Fig. 4:**
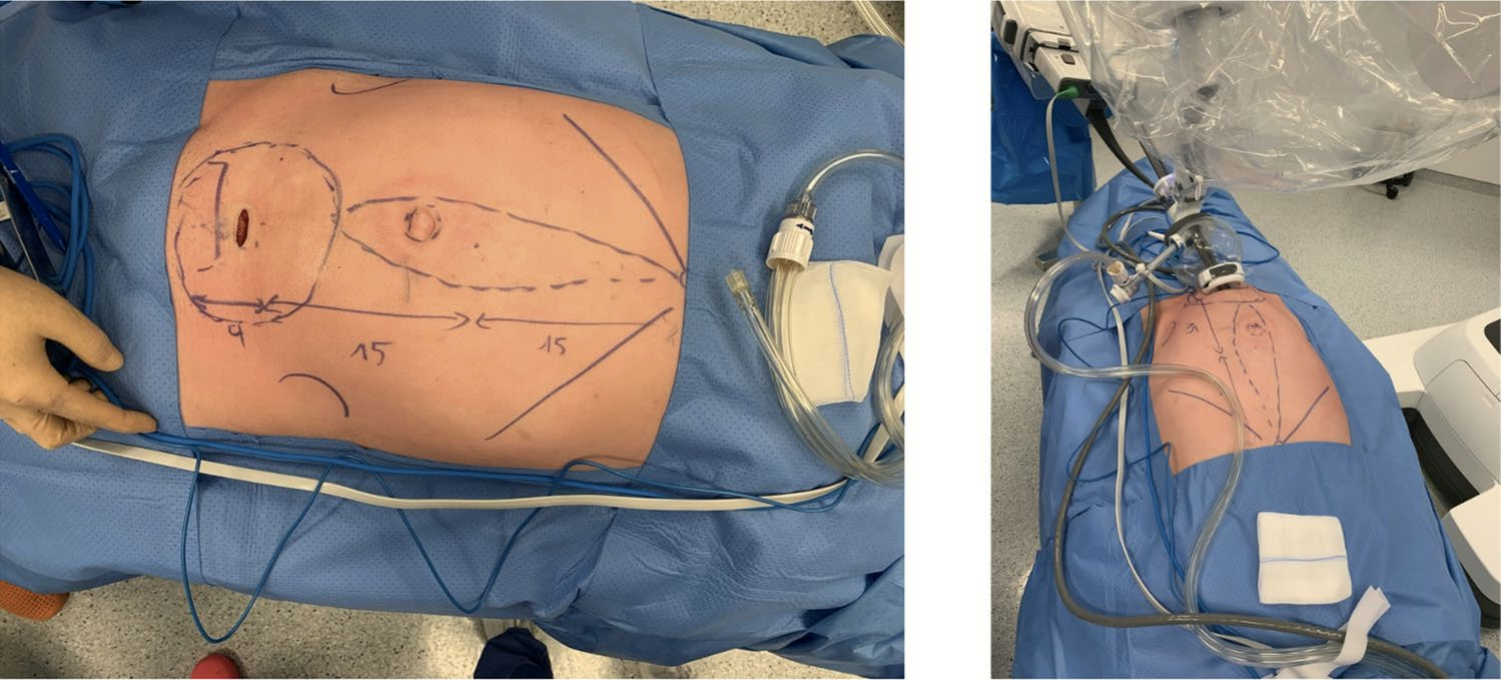
Left: Preoperative planning for single-port suprapubic eTEP, showing abdominal markings and the 27-mm suprapubic incision. Right: The same patient after docking, with the Da Vinci SP system positioned and operating

The preperitoneal space is insufflated to a pressure of 12 mmHg. Throughout the procedure, a fenestrated bipolar forceps is used in the non-dominant hand, while a monopolar curved scissors is operated through the dominant hand. This configuration enables precise dissection and tissue handling during the development of the extraperitoneal working space. The robotic side cart is placed on the right side of the patient, while the single robotic arm is placed in the suprapubic region, with its apex oriented toward the patient’s head. At this stage, the robotic arm is docked to the small access port and the camera and both instruments are inserted through the robotic arm: the camera is positioned inferiorly, the monopolar curved scissors on the right, and the fenestrated bipolar forceps on the left. The space for a fourth instrument, located superiorly, is intentionally left vacant. In this initial phase, the small access port is kept in its expanded configuration to ensure proper deployment of the instruments within the suprapubic region (Fig. 4).

Following access to the preperitoneal space, a decision is made to do either a preperitoneal dissection (in cases of primary non-mesh repair or PeTEP repair) or do a retrorectus dissection (eTEP). In the latter, the posterior rectus sheath is incised bilaterally along the medial edges of the rectus muscles starting at the level of the arcuate lines, leaving the linea alba intact anteriorly. This allows for the development of a retrorectus dissection plane extending laterally to the lateral borders of the rectus sheath. After reduction of the hernia sac, the dissection is carried cranially based on the extent of the hernia defect(s) and the presence and width of diastasis recti. At this stage, when operating in the subxiphoid region, if the instruments reach their limit of extension and maneuverability becomes challenging, the small access port should be collapsed. This adjustment provides approximately 6 additional centimeters of working space, facilitating instrument movement and access.

Cranially, the retrorectus plane is developed up to approximately 6 cm below the xiphoid process, where retrorectus dissection is deliberately halted and the posterior rectus fascia is incised horizontally from medial to lateral. The more cranial dissection in the direction of the xiphoid is thus in the preperitoneal plane. This novel technique, known as the Madrid modification of posterior component separation, is intended to preserve the most cranial neurovascular bundles for the rectus muscles and, probably, to reduce the risk of postoperative bulging due to denervation [14].

The hernia defect(s) and any diastasis are repaired using a running longitudinal slowly absorbable barbed suture (0 V-Loc™, 45 cm, Medtronic) using an imbricating suturing technique often referred to as Inan’s stitch or Geneva stitch. This suturing technique allows for an inwards imbrication of the linea alba minimizing the postoperative ridge of the linea alba seen after a more conventional suturing of the diastasis. In cases where no mesh was used, we added a second longitudinal suture of non-absorbable polypropylene suture (V-Loc 2/0). In the case of retrorectus eTEP, the posterior layer of the rectus sheath beneath the linea alba is also closed if there is no undue tension using the same suture material. A 20 cm × 30 cm non-absorbable polyvinylidene fluoride mesh (DynaMesh®-CICAT) or a 15 × 30 cm self-fixating polyester mesh (Progrip) is then appropriately shaped and placed in the preperitoneal (PeTEP) or retromuscular (eTEP) position, spanning the full extent of the dissected space, in the case of self-fixating mesh with the self-gripping surface oriented toward the rectus muscles for secure fixation. No additional fixation of the mesh is performed and no surgical drains are placed. In one patient (no 22 of our experience), a bilateral TAR was performed and a bigger mesh of 30 × 30 cm was placed.

**Fig. 5:**
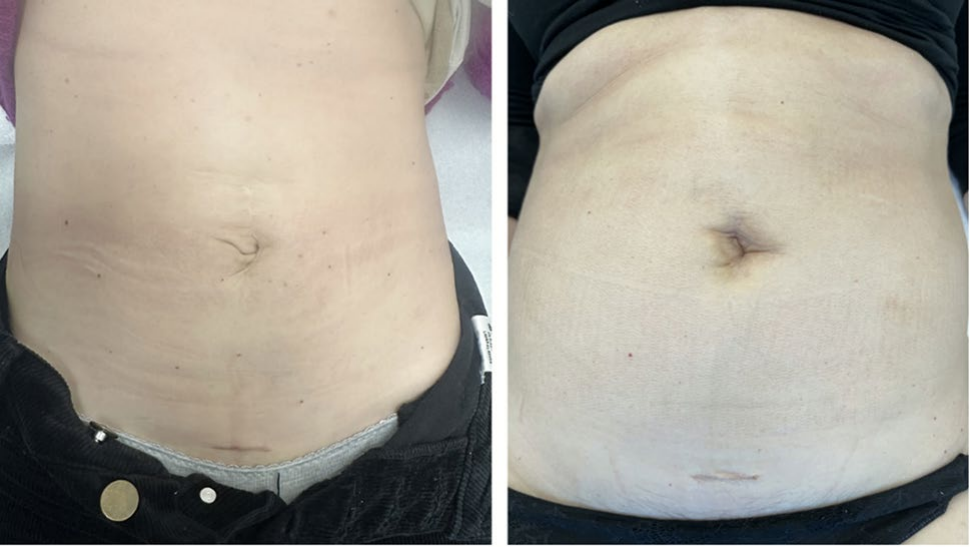
Cosmetic outcomes at 1-month follow-up in two patients who underwent single-port suprapubic eTEP

Any small peritoneal or posterior fascial defects are repaired using either a running barbed suture (3/0 V-Loc™, Medtronic) or interrupted absorbable monofilament sutures (e.g., 3–0 Vicryl™, Ethicon), as appropriate (Fig. 5).

### Outcomes and follow-up

The primary outcome was the incidence of surgical complications, both intraoperatively and during the 1-month postoperative follow-up. Secondary outcomes included the following:

- Operative time
- Hospital length of stay
- Mesh size and plane of implantation

Follow-up was performed via an outpatient clinic visit at 1 month. Complication data were extracted from operative reports, hospital charts, and follow-up documentation.

### Ethics

The study protocol was approved by the ethics committee and the data protection board of the AZ Maria Middelares Gent hospital on 13/08/2025 with reference number RWE.2025.004.

## Results

### Baseline characteristics

A total of 22 patients underwent hernia repair using the Da Vinci SP system during the study period. The majority presented with ventral hernias (*n* = 14), while a subset had inguinal hernias with concomitant umbilical hernias (*n* = 8). Baseline demographic and clinical characteristics, including comorbidities and hernia classifications according to EHS [9], are summarized in Table 1.

**Table 1:** Demographic characteristics, comorbidities, and hernia-related clinical information of the study population

Age (years), median (IQR)		61 (21)
Sex: Male (%)		13 (59%)
BMI (kg/m^2^), median (IQR)		23.8 (5.6)
Presence of relevant comorbidity, *n* (%)		7 (31.8%)
Arterial hypertension, *n* (%)		6 (27.3%)
Cardiac disease, n (%)		4 (18.2%)
Diabetes mellitus, *n* (%)		1 (4.5%)
Hepatic disease, *n* (%)		1 (4.5%)
Pulmonary disease, *n* (%)		2 (9%)
Renal disease, *n* (%)		0 (0)
Active smoker, *n* (%)		2 (9%)
Abdominal aortic aneurysm, *n* (%)		0 (0)
Anticoagulation therapy, *n* (%)		1 (4.5%)
Type of hernia		
	Inguinal, *n* (%)	8 (26.7%)
	Ventral, *n* (%)	14 (63.6%)
	Umbilical (entry site), *n* (%)*	8 (26.7%)
Presence of diastasis recti		
	Yes, *n* (%)	7 (31.8%)
	No, *n* (%)	15 (68.2%)
Localization of inguinal hernia		
	Left, *n* (%)	2 (25%)
	Right, *n* (%)	2 (25%)
	Bilateral, *n* (%)	4 (50%)
Type of ventral hernia		
	Primary, *n* (%)	10 (45.4%)
	Incisional, *n* (%)	12 (54.6%)
Localization of ventral hernia		
	M2	3 (13.6%)
	M3	16 (72.7%)
	M2-3	1 (4.5%)
	M2-3–4	2 (9%)
Length of ventral hernias, cm, median (IQR)		1.6 (1.05)
Width of ventral hernias, cm, median (IQR)		1.7 (1.7)
Width of diastasis recti, cm, median (IQR)		4.1 (0.2)

^*^ Umbilical hernias used as the entry site for the SP Small Access Port during inguinal hernia repair with the Da Vinci SP system

The surgical approach was tailored to the hernia type: a suprapubic single-port access was used for ventral hernias (SP^2^eTEP), whereas in patients with inguinal hernias and concomitant primary umbilical hernia, the SP system was introduced through the umbilical defect. The most frequently employed surgical technique was eTEP (*n* = 14), followed by TAPP (*n* = 5) and TEP (*n* = 3). A range of concomitant procedures, including diastasis recti plication and umbilical hernia repairs, were performed based on anatomical findings. Intraoperative details, such as operative time, mesh dimensions, and wound classification, are presented in Table 2.

**Table 2:** Intraoperative data, including type of surgical procedure, operative time, and related perioperative variables

Type of operation, *n* (%)		
	TAPP	5 (22.7%)
	TEP	3 (13.6)
	eTEP	13 (59%)
	eTEP—TAR	1 (4.5%)
Concomitant repair, *n* (%)		4 (18.2%)
	Umbilical repair (preperitoneal)	2 (9%)
	Diastasis (retromuscular)	10 (45.4%)
	Umbilical repair (retromuscular)	1 (4.5%)
	Diastasis (no mesh)	2 (9%)
	Diastasis (preperitoneal)	1 (4.5%)
	Umbilical repair (primary)	5 (22.7%)
	TAR (retromuscular)	1 (4.5%)
Operative time, inguinal hernia, min, median (IQR)		87 (12)
Operative time, ventral hernia, min, median (IQR)		150 (24)
Intraoperative wound contamination	Class I—Clean	22 (100)
Width of the implanted mesh, cm, median (IQR)		15 (3)
Length of the implanted mesh, cm, median (IQR)		26 (16)
Area of the implanted mesh, cm^2^, median (IQR)		355 (152.5)
Intraoperative surgical complications, *n* (%)		0 (0)
Additional port placement, *n* (%)		0 (0)

No intraoperative complications or conversions were reported. No procedure required patient repositioning or system re-docking. The median length of stay was 19.5 h (IQR = 18 h). All unplanned overnight stays (*n* = 6; 27.3%) were due to postoperative pain requiring inpatient management. Two minor complications were observed at the 1-month follow-up: a seroma and scrotal edema in a patient with previous liver cirrhosis and ascites. No hernia recurrences or readmissions were recorded. A summary of postoperative outcomes is provided in Table 3.

**Table 3:** Postoperative outcomes, including length of hospital stay, complications, recurrences, and readmissions at 1-month follow-up

Duration of stay, hours, median (IQR)	19.5 (18)
Duration of stay, nights, median (IQR)	1 (1)
Non-Planned overnight stay, *n* (%)	6 (27.3)
Intrahospital complications, *n* (%)	0 (0)
Additional visits, before scheduled follow-up, *n* (%)	3 (13.6)
Readmissions, before scheduled follow-up, *n* (%)	0 (0)
Completed follow-up, *n* (%)	22 (100)
Complications at 1 month follow-up, *n* (%)	2 (9%)
Recurrences at 1 month follow-up, *n* (%)	0 (0)

## Discussion

This case series represents, to our knowledge, the first IDEAL stage 1 clinical experience using the Da Vinci SP for abdominal wall surgery, specifically in patients with inguinal hernias with concomitant umbilical defects and in those with midline ventral hernias. The absence of intraoperative complications or conversions, together with only minor short-term morbidity, supports the feasibility and safety of this approach. These findings are consistent with the early reports of robotic single-port surgery and suggest its potential application to increasingly complex abdominal wall reconstructions [4, 10, 15, 15, 16].

Importantly, our experience demonstrates that the same procedures traditionally performed with a multi-port robotic system—TAPP, TEP, eTEP, or TAR—can also be accomplished with the SP platform. The novelty lies not in the surgical techniques themselves but in their execution through a single incision, which may provide cosmetic advantages and reduce the number of trocar sites. Whether these theoretical benefits will translate into improved clinical outcomes cannot be concluded from a small case series and requires confirmation in larger prospective studies.

A notable advantage observed in our series was the collapsible SP small access port. During cranial dissections and midline closure near the xiphoid, the maximum reach obtained (28 cm) was comparable to that of the SP metal cannula (29 cm), but with the added benefit of maintaining full instrument articulation [7]. This adaptability proved critical in completing technically demanding reconstructions, including a bilateral TAR, which underscores the potential breadth of abdominal wall procedures achievable with the SP system.

At the same time, a relevant nuance emerged. The traction and countertraction strength of SP instruments appeared lower than that of multi-port platforms, likely reflecting their dual-jointed design favoring flexibility over force. While this did not compromise safety in our series, it may be a limitation in cases requiring firm traction. More research is needed on this topic and future refinement of SP instrumentation may help address this trade-off in the future. Furthermore, the system requires mastery of additional features such as “cobra camera mode,” which add to the learning curve. Bedside assistance also proved essential: the role of the resident or assistant surgeon was critical in instrument exchanges and troubleshooting, highlighting the need for a well-trained team.

In cases of inguinal hernia repair, our strategy was to initially attempt a TEP approach through the peri-umbilical access. If the peritoneum could be preserved intact during dissection, the procedure was completed as TEP; conversely, in the event of peritoneal breach, the approach was converted to a TAPP repair. It should be acknowledged that the peri-umbilical preperitoneal approach for placement of the SP small access port is technically demanding and presupposes a certain level of expertise and anatomical knowledge. This may not be immediately reproducible for all surgeons. Thus, while feasible and safe in experienced hands, the learning curve for this specific access route may limit its initial generalizability until broader training and standardization are achieved, and its technical demand represents a critical determinant of reproducibility in early experience.

Clinically, short-term outcomes were favorable, with a median length of stay under 24 h and no readmissions, reoperations, or recurrences at 1 month. While encouraging, these results must be interpreted within the limits of a small sample size with a relatively low BMI in our cohort (median 23.8 kg/m^2^), which may limit generalizability, variable surgical techniques, a single-center setting, and short follow-up. Of particular relevance is the ongoing concern about trocar-site hernias in single-incision surgery. Meta-analyses of SILS (Single Incision Laparoscopic Surgery) confirmed a higher incidence of port-site hernias compared to conventional laparoscopy, highlighting the importance of careful fascial closure and long-term surveillance [17]. In our series, this risk was mitigated by strategic patient selection and access planning: in inguinal hernia cases, the umbilical defect itself was used for entry, whereas in ventral hernia repairs, a suprapubic approach was chosen and the final mesh placement overlapped the access site, providing a form of prophylactic reinforcement. Whether the SP platform’s design and this mesh coverage strategy will translate into low rates of trocar-site hernias requires longer follow-up. In fact, given the known risk of trocar-site hernias in single-incision surgery, we plan long-term surveillance of access-site events in this cohort to accurately assess the incidence of port-site hernias over time.

The Da Vinci SP platform is being investigated across surgical fields—general surgery, colorectal surgery, gynecology—with general findings indicating feasibility, safety, and favorable short-term outcomes. A recent systematic review noted that the SP system has been adopted for a variety of procedures, showing faster discharge, shorter operative time, and reduced postoperative pain compared to multi-port robotics [5, 11, 18].

Within the IDEAL framework, our study should be considered an early exploration. Its value lies not in definitive outcome data but in systematically documenting feasibility and highlighting technical nuances. The next steps will require IDEAL Stage 2–3 studies with larger cohorts, standardized techniques, and comparative prospective designs against multi-port and laparoscopic approaches, as well as extended follow-up to assess recurrence, chronic pain, and trocar-site morbidity.

## Conclusion

This preliminary experience confirms the technical feasibility of SP robotic hernia repair for both inguinal and ventral defects, with no intraoperative or early postoperative complications observed. While these findings are encouraging, the comparative benefits, ergonomic implications, and long-term safety of the approach remain to be established. Future multicenter IDEAL Stage 2 studies integrating operative video documentation and patient-reported outcomes will be essential to define the platform’s clinical role.

## Abbreviations

SP: Single Port
EHS: European Hernia Society
IDEAL: Idea Development Exploration Assessment Long-term monitoring
IHR: Inguinal Hernia Repair
TAPP: Trans Abdominal PrePeritoneal
TEP: Totally ExtraPeritoneal
eTEP: Enhanced Totally ExtraPeritoneal
PeTEP: Preperitoneal Enhanced Totally ExtraPeritoneal
TAR: Transversus Abdominis Release
eTEP-TAR: Enhanced Totally ExtraPeritoneal–Transversus Abdominis Release
